# Concatabominations: Identifying Unstable Taxa in Morphological Phylogenetics using a Heuristic Extension to Safe Taxonomic Reduction

**DOI:** 10.1093/sysbio/syu066

**Published:** 2014-09-02

**Authors:** Karen Siu-Ting, Davide Pisani, Christopher J. Creevey, Mark Wilkinson

**Affiliations:** ^1^Department of Biology, National University of Ireland, Maynooth, Co. Kildare, Ireland; ^2^School of Biological Sciences, University of Bristol, Bristol BS8 1TQ, UK; ^3^Department of Life Sciences, The Natural History Museum, London SW7 5BD, UK; and ^4^Institute of Biological, Environmental & Rural Sciences, Aberystwyth University, Aberystwyth SY23 3FG, UK

For a variety of reasons, some phylogenetic data sets are replete with missing entries. Attitudes toward abundant missing data, specifically concerns over its potential to mislead or confound phylogenetic inferences, are varied. Thus, there is a current debate on the impact of missing entries upon the accuracy of phylogenetic inferences (Wiens 2006; Lemmon et al. 2009; Philippe et al. 2011; Wiens and Morrill 2011; Roure et al. 2013). Perhaps less controversial is that individual taxa may sometimes be relatively phylogenetically unstable by virtue of limited data and extensive missing data (e.g., Wilkinson 1996; Sanderson and Shaffer 2002; Wiens 2003; Wilkinson 2003). Wilkinson (1995) developed an approach for diagnosing taxon instability due to missing data *a priori* termed safe taxonomic reduction (STR). STR allows the identification of “rogue” taxa that can be removed from a data set safe in the knowledge that their removal will not impact upon the interrelationships that will be inferred among the remaining taxa under the parsimony criterion. The potential benefits of such deletion are reductions in numbers of optimal trees and run times and better resolved consensus summaries.

STR has been fairly widely used, mainly by paleontologists confronted with relatively incomplete fossil taxa (see Anquetin 2012; Graf 2012; McDonald 2012; for some recent examples), and also in the context of the matrix representation with parsimony (Baum 1992; Ragan 1992) approach to supertree construction (e.g., Cardillo et al. 2004). Nonetheless STR is not always as effective as one might hope (e.g., Mannion et al. 2013). Here, we present a simple heuristic method for identifying potentially unstable taxa that may be useful in cases where STR does not succeed in ameliorating all the problems caused by missing data. We illustrate the approach through application to the saurischian data of Gauthier (1986), which was previously used to illustrate STR and thus is particularly appropriate for demonstrating the ability of the new method to achieve more than STR alone.

## The Method

**Figure 1.**
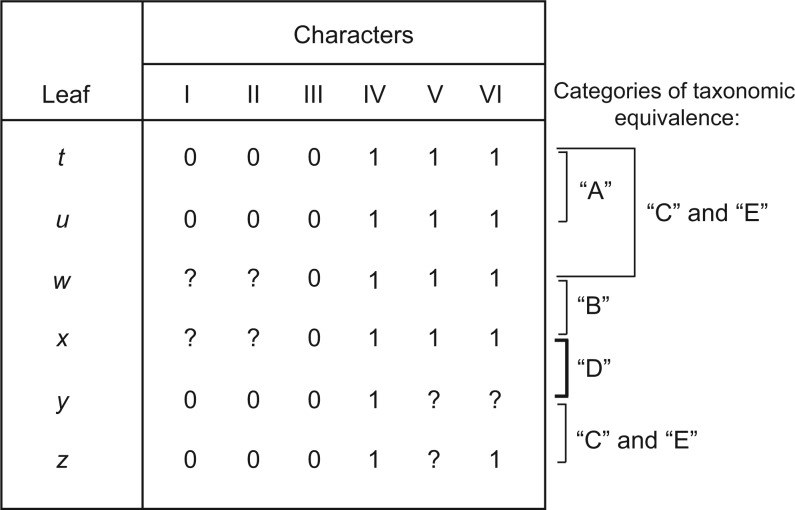
Hypothetical character data illustrating relations of taxonomic equivalence among pairs of taxa (after Wilkinson [1995]) and the categories given in STR. Leaves t and u, which have no missing data and identical character states, are denoted actual equivalents (category A), all the other pairs have some missing data and are denoted potential equivalents. Leaves w and x have identical character data and are denoted symmetric potential equivalents (category B), all the other possible pairs (except t and u, w and x) are asymmetric potential equivalents. Leaves x and y are asymmetric potential equivalents both ways (category D), pairs y and z, and t and w are asymmetric all one way (categories C and E).

STR is based on the understanding that if the character states of a leaf (OTU, terminal, tip) w are a subset of those of a second leaf x (such that w and x have a pairwise-dissimilarity or p-distance of zero) then (i) there exists at least one most parsimonious tree (MPT) in which leaves w and x are a cherry (sister or adjacent taxa) and (ii) removing leaf w will not alter the combinations of character states present in the data, the length of MPTs or relationships inferred among the remaining taxa (Wilkinson 1995). If w is similarly potentially related to multiple other leaves (e.g., to x, y, z, etc.) there will be multiple optimal trees that differ only in the placement of w with x or with y or with z and so on. In such cases, removing w, which adds nothing to a parsimony analysis, can be helpful in reducing numbers of equally optimal trees and improving resolution of strict consensus trees. Leaves that are not demonstrably different with respect to phylogenetically informative characters are called “taxonomic equivalents” (Wilkinson 1995). Figure 1 gives a classification of the sorts of taxonomic equivalence relations that can pertain between pairs of taxa with p-distances of zero.

Sometimes missing (*qua* limited) data seem to be a problem, as evidenced by large numbers of equally optimal trees and poorly resolved consensus trees, but STR is of limited help. In such cases there may be many pairs of leaves with p-distances of zero but, because of the distribution of missing entries, the character states of neither are a proper subset of those of the other (category D, Fig. 1). Wilkinson (1995) called such pairs of leaves “potential taxonomic equivalents that are asymmetric both ways” (we will call them D pairs) and recognized that, in contrast to the other categories of taxonomic equivalence, the deletion of either member of the D pair cannot be guaranteed to be safe *a priori*. The new method we propose augments STR with a ranking of taxa intended to reflect the potential for their deletion to be safe, to substantially reduce numbers of MPTs, and to improve the resolution of strict consensus trees. Unlike STR the method is a heuristic in that the removal of candidate unstable leaves identified *a priori* by the method may not be safe, although it is not difficult to check this *a posteriori*.

The idea behind the new method is very simple. Given any D pair we can ask whether “forcing” these leaves together into a cherry on a parsimony tree would necessitate some homoplasy that is not already evident in the data. If it does not then it seems plausible that the two leaves could go together in some MPT. If one of these leaves has such a relation with many other leaves it seems plausible that this leaf will be unstable in phylogenetic analyses, which may therefore benefit from its removal.

**Figure 2.**
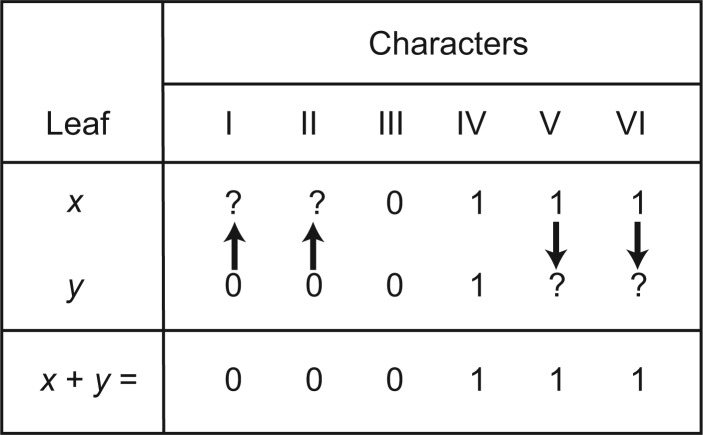
Producing a concatabomination (x+y) for a D pair of taxa with asymmetric potential equivalence both ways. Arrows show how the concatabomination leads to a composite taxon with missing data of each original taxon replaced where possible by data from its pair. In other words, the concatabomination of a D pair is a taxon comprising the union of the character states of the D pair.

Our approach to determining whether homoplasy is increased by forcing leaves to go together makes use of compatibility methods (e.g., Meacham and Estabrook 1985). Two characters are compatible if there is some tree on which they can both fit without any extra steps (homoplasy) and simulations have shown that compatibility decreases as homoplasy increases both for whole matrices (O'Keefe and Wagner 2001) and individual characters (Wagner 2012). We count the total number of character pairs in the data that are incompatible (Le Quesne 1969) and use this as a proxy estimate of homoplasy in the original data. We then combine the data for a D pair of leaves to make what we call a “*concatabomination*” (Fig. 2), add this construct to the original data, and recalculate the pairwise incompatibility. We repeat the latter for each D pair in turn. For each leaf, we define D* as the number of times that leaf contributes to a concatabomination that does not appear to increase homoplasy (i.e., does not increase the number of pairwise character incompatibilities) in the data. We also define, for each leaf, *ABC* as the number of taxonomic equivalents of that leaf in the STR categories A, B, or C (each of which identifies scope for *a priori *safe deletion). Taxa can be ranked based on these individual scores or their sum.

Another way of thinking about this approach is to consider that whereas no individual characters provide evidence against the hypothesis that members of a given D pair are actually the same taxon it is possible that combining their data will reveal incompatibilities (homoplasy) that provide an argument that these leaves do not belong together. Consider a data set in which all pairs of characters are incompatible. In that case adding a concatabomination can never increase the pairwise incompatibility in the matrix irrespective of whether it would entail additional homoplasy or not. In such a case, D* would be maximal for any leaves that contribute to any D pair and provides no basis for discriminating among them. Where the leaves can be ranked based on the sum of their D* and *ABC* scores we envisage users safely deleting any high ranked taxa for which *ABC* is non-zero and then experimentally deleting the taxa with highest D* (or D*+ABC) score to investigate whether this has beneficial impacts (i.e., reduction in numbers of optimal trees, increase in resolution of the strict consensus) while simultaneously checking that the deletion is safe. Removing a taxon is safe precisely when its inclusion or exclusion has no impact upon the inferred relationships of the remaining taxa, that is, when sets of MPTs inferred with the taxon excluded or with the taxon included but subsequently pruned are identical. If tree length is insensitive to the inclusion/exclusion of a taxon this is also a good, though not infallible, indicator that it can be safely deleted (see Wilkinson 1995).

The new method has been implemented into a “*concatabominations pipeline*” in combination with STR that is available at https://bitbucket.org/ksiuting/concatabomination. The pipeline uses modified versions of PerlEQ v.1.0 (Jeffery and Wilkinson's STR software also available at http://www.molekularesystematik.uni-oldenburg.de/en/34011.html) to find all taxonomic equivalents and COMPASS (S. Harris original software also available at http://research.ncl.ac.uk/microbial_eukaryotes/downloads.html) to calculate incompatibility scores. The pipeline tallies the taxonomic equivalents, creates and analyses the concatabominations for every D pair and outputs D* and *ABC* scores of taxa together into a file that can be loaded into Cytoscape (Shannon et al. 2003) to provide a manipulable graphical representation of the results.

## An Empirical Example

**Figure 3.**
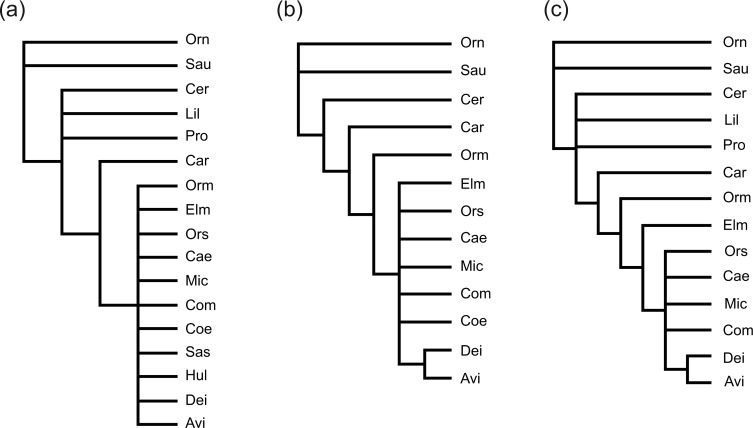
Strict consensus trees of MPTs for the saurischian data of Gauthier (1986) or subsets thereof showing the increase in resolution obtained by deleting taxa. a) the complete data set (no deletions); b) after safe deletion of four taxa identified by STR; c) after deleting the highest ranked taxa identified by the Concatabominations pipeline. For abbreviations used in the trees, refer to Table 1.

We use the Gauthier (1986) morphological cladistic data for saurischians to illustrate the concatabomination approach in practice. This data set is a much cited example of the problems of missing data in paleontological phylogenetics (e.g., Wilkinson 1995; Kearney 2002; Norell and Wheeler 2003), having been previously used to illustrate STR (Wilkinson 1995), and comprising 17 taxa and 84 binary characters with 41% of the entries missing. Missing entries are not randomly distributed in these data but are especially concentrated in some particularly incomplete fossil taxa. Reanalyzed with PAUP* v.4.0b10 (Swofford 2003) with branches collapsed when their maximum lengths are zero, we obtain 832,902 MPTs of 98 steps, the strict consensus of which (Fig. 3a) is disappointingly poorly resolved (with just three splits). Applied to this data set, STR identifies four taxa (*Hulsanpes*, *Liliensternus*, *Procompsognathus*, and *Saurornitholestes*) that can be safely deleted *a priori*. Their deletion results in a substantial reduction in the number of MPTs (to 197, without any change in tree length) and an increase in the resolution (two additional splits) of their corresponding strict consensus tree (Fig. 3b). Note however that this improvement of the strict consensus can be obtained through the deletion of just *Hulsanpes* and *Saurornitholestes*. Although deletions of *Liliensternus *and/or, *Procompsognathus* are both safe and reduce the number of MPTs they are not effective at increasing the resolution of the corresponding strict consensus.

**Table 1.** Results from the concatabominations pipeline analysis of the Gauthier (1986) data set showing numbers of D* and *ABC* scores as well as the percentage of missing entries and abbreviations (Abb.) of taxon names used in the figures.

Taxon	Abb.	% Missing entries	D*	*ABC*	Total
*Hulsanpes*	Hul	81	7	2	9
*Saurornitholestes*	Sas	72	7	1	8
*Coelurus*	Coe	72	5	0	5
*Ornitholestes*	Ors	40	3	0	3
*Compsognathus*	Com	38	3	0	3
*Microvenator*	Mic	67	3	0	3
Ceratosauria	Cer	.0	0	2	2
Deinonychosauria	Dei	6	0	2	2
Caenagnathidae	Cae	33	2	0	2
Elmisauridae	Elm	54	2	0	2
*Procompsognathus*	Pro	64	1	1	2
*Liliensternus*	Lil	48	1	1	2
Ornithomimidae	Orm	8	0	1	1
Ornithischia	Orn	0	0	0	0
Sauropodomorpha	Sau	0	0	0	0
Carnosauria	Car	2	0	0	0
Avialae	Avi	4	0	0	0

**Figure 4.**
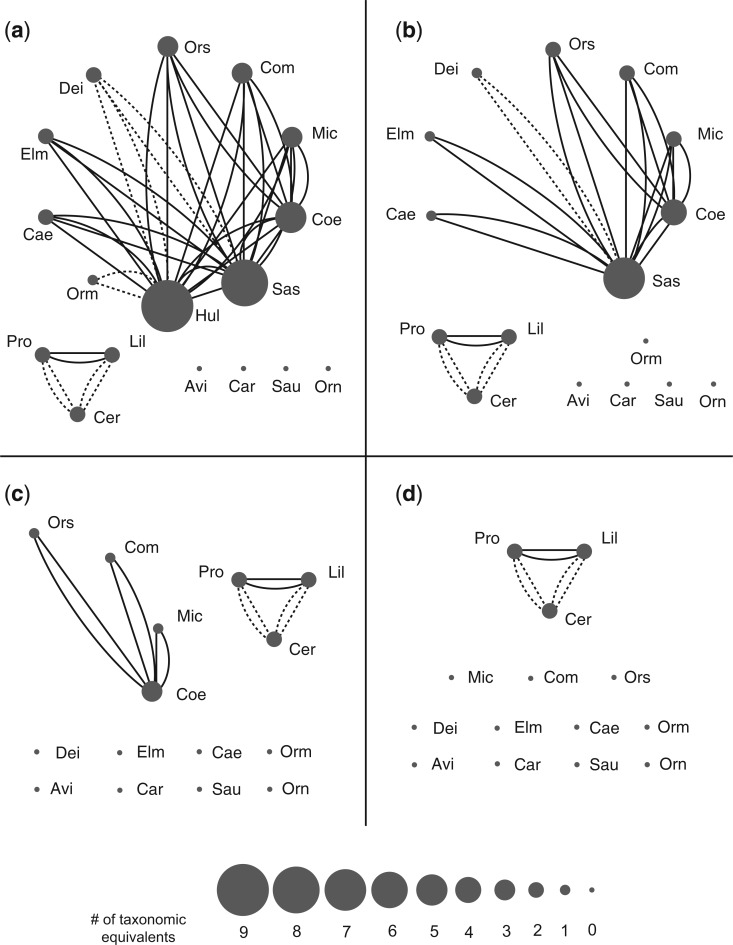
Taxonomic equivalents inferred from the concatabominations pipeline visualized in a network with all taxa (a) and with the successive deletions of *Hulsanpes *(Hul) (b), *Saurornitholestes* (Sas) (c) and *Coelurus *(Coe) (d). Vertices represent taxa and the edges represent a taxonomic equivalence relation existing between the taxa they connect. Vertex size is scaled to represent the number of taxonomic equivalents a taxon has, where the bigger the vertex the more equivalences it has, hence more unstable (see scale at the bottom of figure). Types of equivalences found between taxa are represented by dashed lines (types C and E) and solid lines (type D). For a complete list of abbreviations used for the taxa names refer to Table 1.

Table 1 shows the data obtained from the concatabominations pipeline and Figure 4a provides a graphical representation of the same in Cytoscape with vertices representing leaves and edges connecting pairs that are either (i) taxonomic equivalents in categories A, B, or C (which support safe deletion rules) or (ii) concatabominations that do not increase the pairwise incompatibility of the data. The two leaves with the highest D* (*Hulsanpes* and *Sauronitholestes*) scores are also identified by traditional STR as taxa that can be safely deleted. Deletion of *Hulsanpes* alone reduces the number of MPTs for the remaining data to 45,654 without affecting tree length but does not improve (increase the number of splits in) the corresponding strict consensus. The further deletion of *Saurornitholestes* further reduces the number of MPTs to 2758 and is sufficient to produce all the increased resolution of the consensus (from three to five splits) that can be achieved using traditional STR alone.

Beyond this the two approaches differ. Whereas STR identifies two additional taxa (*Procompsognathus* and *Liliensternus*) that can also be safely deleted, ranking based on D* scores prompts the experimental deletion of *Coelurus*. As already noted, the deletion of *Procompsognathus* and *Liliensternus *reduces the number of MPTs (to 197) but does not further improve the strict consensus. In contrast, deletion of *Coelurus* reduces the number of MPTs to 322 and improves the resolution of the corresponding strict consensus tree by adding an additional split (Fig. 3c). Deletion of *Coelurus* does not change MPT length and the set of trees produced from the data after its deletion is identical to the trees produced with it included but from which it has been pruned. Thus, we can be confident that the deletion of *Coelurus* is safe although it was not identified *a priori* as such by traditional STR.

We find using a graphical representation of the concatabominations pipeline output (Fig. 4), in which the degree of each vertex (leaf) represents the sum of the D* and *ABC* scores, to be very useful for visualizing the potential equivalence relations among the taxa and especially useful in showing how these change with the successive removal of taxa (Fig. 4b–d). Disconnected components in the graph also help identify independent sets of taxonomic equivalents (e.g., the small set including *Procompsognathus* and *Liliensternus* and the main set that contains *Hulsanpes* and *Saurornitholestes*). Rather than deleting taxa in the order suggested by the initial ranking of their scores, it makes more sense to recalculate the scores and re-rank the taxa after each deletion and this is perhaps most easily accomplished in Cytoscape. Note that after the deletion of *Coelurus* (Fig. 4d) all the taxa that were previously connected in the main set are now unconnected indicating no further potential taxonomic equivalence among those taxa.

In this example, the analysis can stop at this point because although additional safe deletions may be possible they cannot be expected to lead to sufficiently reduced numbers of MPTs such as to lead to additional splits in the corresponding strict consensus. Hence we find, *a posteriori*, that the deletions of two other taxa (*Ornitholestes* and *Microvenator*) are also safe but do not lead to any improvements of the strict consensus and are therefore quite unnecessary. More generally, as the graph becomes more and more disconnected the method will offer fewer and fewer candidates for experimental deletion.

## Discussion

Since its introduction, STR has been adopted by many phylogenetic paleontologists as a means of identifying relatively unstable rogue taxa that can obfuscate what analyses of the data can tell us about phylogenetic relationships of other relatively more stable taxa, with varying degrees of success. It has also been applied in some supertree studies that employ matrix representations (pseudocharacter encodings) of input trees. One undoubted attraction of STR is that a taxon is deleted *a priori *only if we are certain that this deletion cannot impact upon the relationships inferred among the remaining taxa. Thus, it is not like throwing away data that could have an impact on the result and is consistent with a “total evidence” philosophy.

Taxon deletion is safe whenever the sets of trees produced *a posteriori* by (i) excluding the taxon from the data and (ii) pruning it from MPTs inferred with it included are identical. In any particular case there may be useful safe taxon deletions that are not identified *a priori* using STR. Our concatabomination approach is motivated by the desire to extend or augment STR by discovering these. It is a heuristic for identifying candidate rogue taxa, the deletion of which can only be confirmed as safe *a posteriori*. It is heuristic in that there are conditions where (i) it might suggest taxa that are not safe to delete (hence the suggestion to confirm safety *a posteriori*), (ii) it may fail to suggest taxa that could be safely deleted, and (iii) the order in which taxa are recommended as candidates for experimental deletion may depend upon what taxa have already been deleted and how any ties have been broken. One such condition is when the original data matrix already has a maximal incompatibility score, and hence substitution of each pair of taxa for their concatabomination cannot result in an increased incompatibility score.

It is worth noting that even the “safe” removal of taxa might impact upon branch length estimation in parametric, model-based phylogenetics and that in stratocladistics (Fisher 2008) where deleting potential equivalents would be counterproductive if they are from different time intervals. Identification and safe removal of taxonomic equivalents might also be worth considering in analyses of disparity using cladistic data, and in the haplotype inference problem (Clark 1990; Gusfield 2004) if missing data lead to multiple optimal solutions, but any use and impact on these areas will require further study.

The example data set we used to illustrate the approach served also in the development of STR and might be considered fairly well studied and understood. Thus, we were surprised when application of the concatabomination approach to these data led to such a clear cut improvement over what was achievable with STR alone. The example nicely illustrates how the approach can successfully lead to additional safe taxon deletions that improve the resolution of the strict consensus tree and our understanding of what phylogenetic hypotheses are supported by the parsimonious interpretation of the data. Although the approach is heuristic, we expect that highly ranked taxa that it identifies will in practice be the ones that most likely can be safely deleted (because there is no evidence of unique combinations of character states to suggest deletion will be unsafe) while usefully reducing the number of MPTs (because they have multiple potential equivalences corresponding to multiple positions in the MPTs).

Although not necessary, we find the graphical representation of the results, with each taxon a vertex and edges representing potential equivalence, and the manipulation it enables to be particularly helpful. As highly connected, potentially unstable, taxa are deleted any changes in the degree of the remaining vertices and of their relative rankings will be apparent. Natural stopping points for experimental deletion are when formerly connected clusters of taxa completely separate or when connected taxa cannot be safely deleted or their safe deletion does not improve the consensus.

Recently, there has been growing interest in the detection of rogue taxa in large-scale phylogenetics mostly using purely *a posteriori* approaches (Aberer and Stamatakis 2011; Pattengale et al. 2011). Concatabominations, which sits somewhat between the pure *a priori* approach of STR and purely *a posteriori* approaches such as leaf stability (Thorley and Wilkinson 1999) or reduced consensus (Wilkinson 1994), offers another approach to this problem. That this approach can be applied to matrix representations of trees highlights its potential in diagnosing the often serious problem of ineffective overlap in broad phylogenomic (multi-gene) studies and in supertree construction (Wilkinson and Cotton 2006; Sanderson et al. 2011).

## Funding

This work was supported by the Biotechnology and Biological Sciences Research Council grant [BB/K007440/1 to M.W.] and by an EMBARK scholarship from the Irish Research Council awarded to K.S.
